# Pneumatically‐Actuated Liquid Metal‐Based Frequency Reconfigurable Antenna

**DOI:** 10.1002/advs.202512996

**Published:** 2025-12-12

**Authors:** Yiwen Song, Aditya Bharambe, Dinesh K. Patel, Barbara Zhuo, Mason Zadan, Carmel Majidi, Swarun Kumar

**Affiliations:** ^1^ Department of Electrical and Computer Engineering Carnegie Mellon University 5000 Forbes Avenue Pittsburgh PA 15213 USA; ^2^ Department of Mechanical Engineering Carnegie Mellon University 5000 Forbes Avenue Pittsburgh PA 15213 USA; ^3^ Current Affiliation: Koch Institute for Integrative Cancer Research, Massachusetts Institute of Technology, 77 Massachusetts Avenue, Cambridge, MA 02139, USA

**Keywords:** antenna, liquid metal, pneumatic actuator, soft robot

## Abstract

Software‐defined radios and cognitive radios can switch their operating frequency across a wide range. Although analog and digital circuits enable cross‐frequency operation of much of the RF frontend, there is a lack of practical antennas that are frequency‐reconfigurable over such a wide range. Prior rigid antennas are either narrow‐band with high radiation efficiency, or wide‐band with low radiation efficiency. This study presents a soft, stretchable, compact, omnidirectional liquid metal composite antenna system to enable high radiation efficiency when reconfiguring to different frequencies across a wide band. On the hardware front, an extendable multi‐branch stretchable liquid metal based antenna geometry is optimized that preserves high efficiency when switching across a wide range of frequencies. On the software front, a control algorithm is incorporated that ensures that the antenna automatically configures to the optimal received signal strength. The antenna design enables a strain limit of 33% for two independent actuatable branches, further enabling a switchable operating frequency range across 1–5 GHz. Experimental characterization demonstrates that PASTA reduces the radiation loss by 10–20 dB over the operating frequency range compared to state‐of‐the‐art rigid and flexible antennas.

## Graphical abstract

A look at a flexible, frequency‐tuning antenna

- **A Soft Solution**: Introducing a new antenna design made with liquid metal composites.
- **A Full Package**: Combining flexible antennas with pneumatic actuators.
- **End‐to‐End Control**: Automatic reconfiguration according to signal strength.
- **Putting It to the Test**: A summary of the antenna's performance and capabilities.
- **Future Possibilities**: Exploring potential applications for this type of technology.

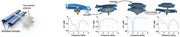

## 1 Introduction

Software‐defined radios (SDRs) are radios that can be software‐programmed to operate in a wide range of frequencies, such as 100–5000 MHz.^[^ ^1^ ^]^ They have wide‐ranging applications from wireless systems research,^[^ ^2^, ^3^ ^]^ education,^[^ ^4^, ^5^ ^]^ and various specialized domains including search‐and‐rescue,^[^ ^6^ ^]^ astronomy,^[^ ^7^ ^]^ and medicine.^[^ ^8^ ^]^ SDRs are a critical component for wireless systems ranging from cognitive radio,^[^ ^9^, ^10^ ^]^ RF backscatter^[^ ^11^, ^12^, ^13^ ^]^ to wireless sensing^[^ ^14^, ^15^ ^]^ and imaging.^[^ ^16^, ^17^ ^]^ Much of this work relies on the high degree of reconfigurability of SDRs, where they allow the transmission of arbitrary raw complex wireless signals that can be modulated at a wide range of carrier frequencies. However, a key component of SDRs evades direct programmability – its physical antenna. Although commercial SDRs support switching across a large range of frequencies, the rigid antennas attached to them are usually constrained to work in a limited frequency range. Traditionally, rigid wideband antennas are designed by selecting a physical structure—called a resonator—that naturally supports standing electromagnetic (EM) waves at multiple frequencies. These standing waves are constructed by EM wave reflection within the antenna geometry, allowing the antenna to efficiently transmit or receive signals over a wide range of frequencies.^[^ ^18^, ^19^ ^]^ However, rigid wideband antennas with moving or electronically‐switchable rigid parts have also been developed, such as telescopic antennas^[^ ^20^, ^21^ ^]^ and software‐defined antennas.^[^ ^22^ ^]^ While rigid wideband antennas are bulky and highly directional, those ones with switchable parts either use a large motor module to actuate the antennas or have a large array of antennas, making them unfriendly for ubiquitous applications.

The development of flexible electronics^[^ ^23^, ^24^ ^]^ and stretchable conductors^[^ ^25^, ^26^ ^]^ has opened opportunities for flexible and reconfigurable antenna designs.^[^ ^27^ ^]^ To show the potential of utilizing soft materials and electronics into next‐generation flexible antennas, past works have replicated traditional narrow‐band antenna geometries into soft, flexible antennas, such as dipole antennas,^[^ ^28^ ^]^ patch antennas,^[^ ^29^, ^30^, ^31^ ^]^ and slot antennas.^[^ ^32^ ^]^ The principle of such antennas is that, by changing the length of the resonating element, the operating wavelength of the antennas changes accordingly. However, since soft actuators usually allow a maximum of 100% (or –50%) strain, the flexible antennas based on traditional geometries only provide limited frequency reconfigurability, e.g., within 1–2 GHz before becoming unrealistic in size. For example, past papers have evaluated the performance and shown improvement for multiple microwave bands spanning from sub‐GHz to over 10 GHz^[^ ^33^, ^34^, ^35^, ^36^, ^37^ ^]^ when compared to traditional rigid antennas. The shape deformation studied in these works includes bending, stretching, and compression, with the strain ranging from a few percent to more than 100%.

While these previous efforts in soft antennas for frequency reconfigurability have been promising, several questions remain to be answered. These include the following key: (i) designing reconfigurable antennas to cover more frequencies at the same form factor; (ii) packaging the actuator with the antenna to make it lightweight, soft, and robust; (iii) controlling the antenna to adapt to its operating frequency automatically according to signal feedback. This paper introduces Pneumatically Actuated Software‐Tunable Antenna (PASTA), which is an approach that addresses key challenges the creation of soft, actuatable, frequency‐reconfigurable antennas.

First, PASTA introduces antenna geometry that supports multiple actuatable parts, allowing the antenna to be configured across a wider frequency range. PASTA achieves this by introducing a flexible two‐branch monopole antenna structure and an optimization method that impedance‐matches the antenna toward the 1–5 GHz wide operating frequency bands. The two branches of the antenna offer the antenna with a wide range of operating frequencies with a small form factor of 30×90 mm. PASTA operates in the range from 1 to 5 GHz, which covers most frequencies of typical SDRs and communication systems, including the cellular (5G), WiFi, and amateur radio frequency bands. The antenna geometry is also optimized toward better impedance matching during the actuation process, which translates to higher radiation efficiency. Second, PASTA proposes a lightweight antenna packaging with soft pneumatic McKibben actuators. We 3D print the antenna on silicon rubber substrates with highly conductive, stretchable inks.^[^ ^38^ ^]^ Since rigid actuators such as step motors and servo‐motors are incompatible with the mechanical compliance and elasticity of PASTA, we package the McKibben actuators with the antenna branches to make the antenna fully soft and ensure fast actuation. McKibben actuators are soft, compact, cost‐efficient, and lightweight, making them suitable for building ubiquitous soft antenna systems. By selecting McKibben actuators, we are also able to reach a higher strain in the same area when compared to other soft actuators such as liquid crystalline elastomers,^[^ ^39^, ^40^, ^41^ ^]^ shape‐memory alloys,^[^ ^42^, ^43^ ^]^ and dielectric elastomers.^[^ ^44^, ^45^, ^46^ ^]^ Third, PASTA designs a control algorithm that automatically configures the antenna toward the current operating frequency according to received signal strength measurements. PASTA is first configured to the working frequency according to an offline chart, and then uses a PID algorithm to keep configuring itself to higher received signal strength. The two‐stage closed‐loop actuation control algorithm achieves a real‐time fast configuration within 1 second. In summary, the paper proposes an interdisciplinary systematic design for flexible antennas, which advances past flexible antenna works toward practical applications in antenna‐actuation co‐design and system packaging with 3D‐printable antenna structures, pneumatic actuators, and feedback‐loop actuation control.

## 2 Results

PASTA explores the design of a shape‐shifting programmable antenna that resonates at desired frequencies. Antennas are tuned to specific resonating frequencies because standing waves at such frequencies form on the antenna. The formation of standing waves is determined by the electrical length of the antenna. Therefore, a shape change that changes the electrical length of the antenna influences its operating frequency. To make a large transition of operating frequency happen on a small form‐factor antenna, this paper combines innovations in three parts: (1) The antenna design proposes a dual‐branch structure for flexible antennas to enable double frequency bands with a small form factor. (2) A 3D printing‐based fabrication process enables rapid, robust, and low‐cost fabrication. (3) A two‐stage control process allows the antenna to be quickly configured to the desired operating frequency.

### 2.1 Design and Fabrication of the Reconfigurable Antenna

PASTA proposes a unique antenna design pipeline optimized for flexible antennas. For antennas, two types of shape changes affect the operating frequency the most: *stretch/compression* and *curvature*. In PASTA, we focus on changing the antenna operating frequency by stretching. We adopt a uni‐axial stretching model for thin films,^[^ ^47^, ^48^ ^]^ where one axis is actuated to control the operating frequency of the antenna. The silicone rubber substrate is treated as a non‐compressible material. Assuming that the *x* axis is stretched by a factor of μ, i.e., *x*′ = (1 + μ)*x*_0_, it follows from material incompressibility that the antenna along the *y* axis is compressed by a factor of 1/(1 + μ).

**Figure 1:**
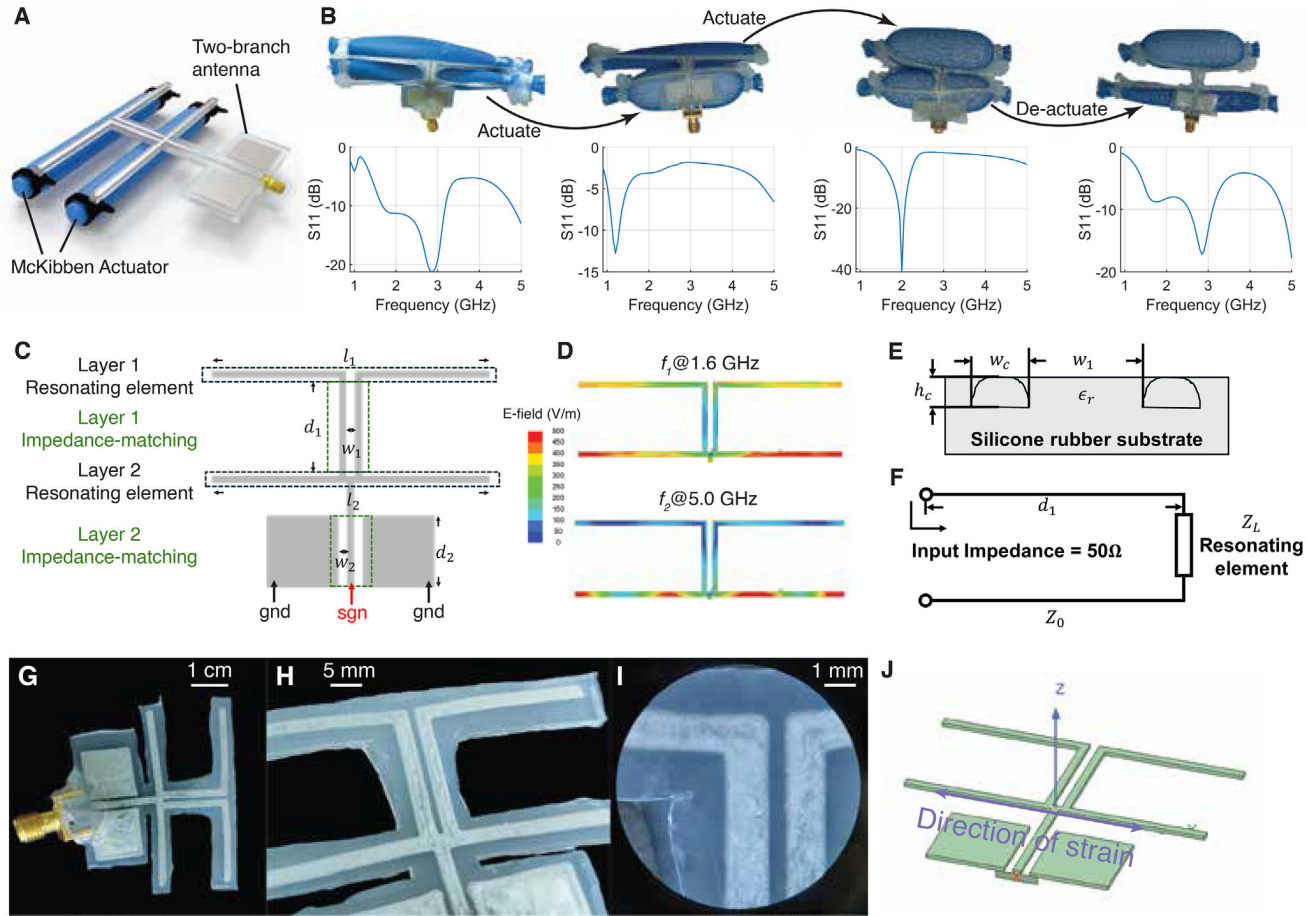
Design and fabrication of a soft, reconfigurable antenna. A) Two‐branch antenna with McKibben actuators that can be configured for a wide range of frequencies. B) Each branch of the antenna can be actuated to achieve different operating frequencies. C) Impedance matching for dual‐branch antennas. The branch lengths *l*_1_ and *l*_2_ are changed during actuation. To best impedance‐match each component, we design impedance‐matching components by parallel lines controlled by *d*_1_, *w*_1_, *d*_2_, *w*_2_. D) Electric field intensity of the antenna branches of 0% strain (55 mm) at two different frequencies. At a low frequency (1.6 GHz), the resonance appears at both branches. At a high frequency (5.0 GHz), the resonance appears at only the lower branch. E) Impedance matching. The cross‐section of the conductive traces (dashed) inside silicone rubber (grey). *w*_*c*_ and *h*_*c*_ are restricted by printing, ϵ_*r*_ represents the relative permittivity of silicone rubber. F) The characteristic impedance *Z*_0_ (controlled by *w*_1_) and the length of the line *d*_1_ can be optimized. Antenna prototype. G) Fabricated antenna with the attached SMA connector. H) Detailed printed trace on the antenna. I) Microscopy image of a stretched antenna. J) A model of PASTA's antenna and the direction of strain.

We seek a design that allows for greater mechanical compliance and elastic deformability, which lead us to adopt a single‐layer extended monopole antenna design as described in **Figure** **1**A. Such deformability allows for a larger operating frequency range, in which the frequency can be controlled using a modest tensile force. This monopole antenna design allows the antenna to be omnidirectional, which allows the antenna to work without the requirement of mechanical steering. When stretching the monopole antennas by a factor of μ, the operating frequency decreases to *f* ′ = *f*_0_/(1 + μ). For a flexible monopole antenna to operate in the 1–5 GHz range, it needs to be stretched to 5× the original length, which leads to the bulkiness of the flexible system. To tackle the challenge, as shown in Figure 1B, to enable the flexible antenna system to be reconfigured to work on a wide range of frequencies, PASTA proposes an extendable dual‐branch antenna design that compliments two independent actuation processes such that the antenna works and configures two frequencies at the same time. The basic idea of the dual‐branch design is that each branch on the antenna is designed to be responsible for a different range of frequencies, and therefore, by selectively actuating each branch, the antenna can be configured to a wide range of frequencies.

PASTA utilizes a fixed impedance matching element across frequencies for each branch. Although a floating matching circuit would theoretically be optimal since the resonating elements have different impedances when actuated, adding too many electronically or mechanically controlled lumped elements can cause the antenna to be bulky and rigid. Moreover, adding lumped elements to the flexible conductive patterns that construct the antenna reduces robustness due to the lack of stable connection at the interfaces of rigid and flexible components when large shape changes occur. Instead, although a fixed impedance matching element that is achieved by 3D printing conductive traces is less optimal, it enables low‐cost and robust antennas that can be massively fabricated. Therefore, we optimize parallel transmission line patterns to improve performance across multiple actuation states. However, the antenna efficiency is not a convex function with respect to the variables. Therefore, it is computationally hard to find the optimal variable combination to maximize the antenna efficiency across frequencies by brute force search, because in each iteration of the optimization, multiple rounds of frequency sweeps need to be performed to find the entire S11 parameter with regard to all antenna actuation states, which can take hours. To reduce the computational cost, we design an optimization flow that reduces the number of required rounds for simulation. We follow a layer‐by‐layer approach for each component. For each layer, we first use a model‐based approach to find a set of parameters that match the antenna impedance, which is simulation‐free. We then combine the parameters and use gradient‐based refinement to optimize the result, which only requires a few simulation iterations to find the optimal result, since the parameter search space is narrowed by the model computation. Figure 1C shows the optimization flow for our 2‐branch antenna. It can be extended to antennas with more layers. Figure 1D shows when operating at higher frequencies, the lower branch provides higher resonance. When operating at lower frequencies, as a larger electrical length is required, both branches are resonant, thus providing twice the length of a single branch.

The impedance matching process is performed top‐down. We start from the first layer of the resonating element. A parallel line is used for the impedance matching via the control of spacing *w*_1_ and length *d*_1_. Figure 1E shows the cross‐section of the parallel line, where two conductive traces separated by *w*_1_ are buried inside a silicone rubber substrate of relative permittivity ϵ_*r*_. The width of the printed line is limited by fabrication, which we set at 0.5 mm. The characteristic impedance *Z*_0_ can be approximated as follows^[^ ^49^ ^]^

(1) $$Z_{0}=\frac{87}{\sqrt{\varepsilon_{r}+1.41}\ln\left[\frac{5.98w_{1}}{0.8h_{c}+w_{c}}\right]}\;.$$

After approximating the characteristic impedance of the parallel line, we can alter the length of the parallel line to control the input impedance relative to the load of the resonating element *Z*_*L*_, as shown in Figure 1F. The impedance matching problem of a *single frequency* can be formulated as follows:

(2) $$\min_{w_{1},d_{1}}\;{\left|50\Omega -Z_{0}\left(\frac{Z_{L}+jZ_{0}\tan\left(\beta d_{1}\right)}{Z_{0}+jZ_{L}\tan\left(\beta d_{1}\right)}\right)\right|}^{2}\;.$$

However, solving Eq. (2) can not give the optimal matching result. The resonating element is actuated and its operating frequency and impedance change correspondingly. Therefore, we solve the matching problem to find a set of parameter values that optimize the impedance matching throughout the overall actuation process.

We first evaluate the change in impedance *Z*_*L*_ when the length *l* is stretched from *l*_0_ to (1 + μ)*l*_0_ by simulation. *N* multiple stretching factors are sampled uniformly and their corresponding frequencies and impedances are recorded, denoted by *f*_1_, *f*_2_, ⋅⋅⋅, *f*_*N*_ and *Z*_1_, *Z*_2_, ⋅⋅⋅, *Z*_*N*_. We calculate the impedance matching parameter *w*_1_, *d*_1_ that best matches all the possible impedance values. The problem can be formulated as:

(3) $$\min_{w_{1},d_{1}}\sum_{n=1}^{N}{\left|50\Omega -Z_{0}\left(\frac{Z_{n}+jZ_{0}\tan\left(\beta d_{1}\right)}{Z_{0}+jZ_{n}\tan\left(\beta d_{1}\right)}\right)\right|}^{2}$$

The optimization problem is non‐convex. We approximate the solution by performing gradient descent from multiple random initial points. As the number of initial points is large enough, the gradient descent algorithm is likely to provide the optimal solution.^[^ ^50^ ^]^ Since solving the optimization problem is quick with two variables, we use 100 starting points uniformly sampled between the search space of *w*_1_ and *d*_1_. The second‐layer impedance matching can be solved similarly by simulating the impedance of the second layer.

Despite the result provided by the optimization, several factors can lead to an error. First, Eq. (1) considers a microstrip line, while we consider the impedance of two parallel strip lines. Moreover, the formula is acquired through fitting the experimental results, which are not theoretically proven under all frequencies. These two factors can cause Eq. (1) to incur errors during impedance estimation.

Considering the errors from the optimization process, we need to refine the impedance matching. To this end, note that S11 values represent the amount of power that is reflected back from the input port. The lower the S11 values are, the better the antenna efficiency is. We therefore aim to minimize the maximum S11 value at the input port of the antenna at uniformly sampled actuation points μ_1_, μ_2_, ⋅⋅⋅, µ_*M*_ where 0 = μ_1_ < μ_2_ < ⋅⋅⋅ < µ_*M*_ = µ_max _. We take the S11 values at their working frequencies *f*_*w*_. The working frequencies are defined as the peak of the convex S11 function with regard to frequency, which is the minimum S11 point at this actuation state, i.e.,

(4) $$f_{w}\left(\mu \right)=\arg\min_{f}S11\left(f,\mu \right)\;.$$

During the refinement phase, we simultaneously optimize the four parameters *w*_1_, *d*_1_, *w*_2_, *d*_2_ shown in Figure 1E, which can be formulated as follows

(5) $$\min_{w_{1},d_{1},w_{2},d_{2}}\max_{1\leq m\leq M}\min_{f_{\min}\leq f\leq f_{\max}}S11\left(f,\mu_{m}\right).$$

**Figure 2:**
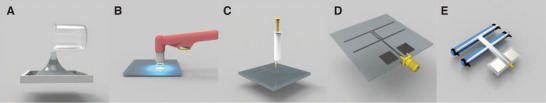
Fabrication of PASTA. A) Molded silicone rubber substrate. B) The substrate is then plasma‐treated to clean and increase adhesion to the conductive layer. C) We 3D printed the antenna on the substrate using Ag‐EGaIn‐SIS ink. D) An SMA connector is added to the antenna with a sealing layer of silicone elastomer cured on top. E) The antenna is cut and adhered to the McKibben actuator.

During the optimization phase, we set a small search space as ±10% offset from the initial point. The reason why we set a small search space is due to the unpreventable fabrication offset and to reduce the computational cost. Through the two‐step optimization, the computationally hard impedance matching problem across a large frequency range and a large actuation range is simplified. Figure 1G–I shows a fabricated prototype of PASTA, while **Figure** **2** shows the fabrication process. Liquid metal droplets can be seen at the top layer of the printed conductive pattern. The liquid metal droplets maintain the trace conductivity even when it is stretched by over 100%.^[^ ^38^ ^]^

With the fabricated antenna prototype, we demonstrate that PASTA's antenna is of small and compact form factor. **Table** **1** compares the form factor and the corresponding operating frequency range with the common state‐of‐the‐art antennas. It can be seen that PASTA has a good balance between form factor and operating frequency range. Compared with state‐of‐the‐art flexible antennas, PASTA achieves a similar form factor but 2× frequency range. Compared with rigid antennas, PASTA achieves a smaller frequency range, but a much smaller form factor (0.2×). We also compare the mass of the listed antenna systems to show the advantage of PASTA to be lightweight. First, when comparing the bulk mass of the antenna itself, the mass of PASTA's antenna is only 10 grams. A flexible patch antenna requires a thicker substrate and two‐layered liquid metal, which increases the antenna mass to 35 grams. Rigid antennas such as Vivaldi antennas and horn antennas are as heavy as 130–1300 grams. To compare the overall mass, when adding the mass of the antenna to the pneumatic actuation system, including the actuator, a pump, and valves, the overall system mass of PASTA goes to around 100 grams, which is still lightweight and comparable to rigid antennas. Finally, when it comes to a rough estimation of antenna performance, we characterize the performance in return loss. PASTA achieves larger than 10 dB return loss in almost all frequencies when properly configured, within the 1‐5 GHz designed operation range. It is comparable to or superior to other soft antennas, while increasing the overall reconfigurable bandwidth. It is also comparable to or superior to rigid antenna systems. PASTA's design is also extendable to achieve a larger operating frequency range if needed by adding more branches and corresponding impedance layers.

**Table 1:** Form factor comparison of state‐of‐the‐art flexible antennas, wideband antennas, and PASTA. (F) refers to flexible antenna, (R) refers to rigid antenna. (A) refers to mass of only the antenna. (S) refers to mass of the antenna plus the actuator and actuation system (only applies for flexible antennas).

Antenna	R/F	Freq [GHz]	H×W×T [mm]	Mass [A] [grams]	Mass [S] [grams]	Return Loss [dB]	Refs.
Dipole	F	0.75 – 2	(40 – 148) × ∼20 × ∼20^a)^	∼10 – 100^a)^	∼100 – 200^a)^	>10 dB	[28]
Patch	F	3 – 5^b)^	40 × (40 – 80) × 6^b)^	35^b)^	125^b)^	>7.5 dB^b)^	[51]
Vivaldi	R	0.9 – 12	165 × 230× 3	130	N/A	>7.5 dB	[52]
Horn	R	5 – 6	261 × 214 × 217	1300	N/A	>20 dB	[53]
PASTA	F	1 – 5	30 × (55 – 90) × 6	10	100	>10 dB	This work

a) Estimation is acquired through figures and Supporting Information of paper.

b) The result is reproduced using the same method as in the paper, the number is measured without the actuator.

### 2.2 Antenna Packaging

The actuation performance of the antenna is critical to providing a high frequency‐tuning range, rapid configuration, and accurate control. We aim to find soft, low‐cost, and endurable actuators so that the antenna can be applied to a broad range of use cases without introducing bulk or rigidity. Traditional actuators such as step motors and servo motors are accurate, but are hard‐cased and can be bulky. Therefore, we choose soft actuators that can be integrated with the antenna and distributed over the two branches to achieve decoupled motion. These include shape‐memory alloys,^[^ ^42^, ^43^ ^]^ thermally responsive liquid crystal elastomers,^[^ ^39^, ^40^, ^41^ ^]^ pneumatic artificial muscle (PAM) actuators,^[^ ^54^, ^55^, ^56^ ^]^ voltage‐controlled dielectric elastomer actuator,^[^ ^44^, ^45^, ^46^ ^]^ and chemo‐mechanical actuators that are actuated by chemical reactions.^[^ ^57^, ^58^, ^59^ ^]^ One candidate that we considered is heat‐based actuators like shape‐memory alloys (SMAs) and liquid crystalline elastomers (LCEs), which respond to changes in temperature.^[^ ^38^, ^60^, ^61^, ^62^, ^63^ ^]^ Although they have a good contraction ratio and small form factor, the actuation and relaxation speed of these actuators is very slow due to the bad thermal conduction between the actuator and air. Moreover, they need a consistent power supply to keep a contracting state, which makes them power‐inefficient as they consume much more power than the antenna itself. Dielectric elastomer actuators are a promising alternative that uses high voltage to induce contraction through electrostatic attraction.^[^ ^64^, ^65^, ^66^, ^67^ ^]^ However, the contraction ratio for dielectric actuators is small, thus limiting the maximum deformation and allowable frequency range for the antenna. Furthermore, the conductive layers in the dielectric actuator cause interference with the antenna as it changes shape, and can lead to performance degradation. Finally, ferromagnetic actuators are a popular approach in soft robotics. However, actuation depends on the use of a high‐power electric magnet, which can be power‐hungry and interfere with antenna transmission.

These so‐called “McKibben actuators” expand and contract when inflated, and have a 1:3 contraction ratio when fully actuated. For PASTA, we selected McKibben actuators, which are a type of pneumatic artificial muscle that provides a good tradeoff between our various requirements. Not only are McKibben actuators entirely soft, but they also have a relatively large contraction ratio (∼40%) which provides a high frequency‐tuning range. Their actuation is fast even with a low‐power pump (∼5 seconds), and when a valve is closed, the actuator can remain in a stable position for a long time. The speed of relaxation is controlled by removing compressed air using a release valve, a process that can be as fast or faster than actuation. McKibben actuators can be made at low cost using commercial off‐the‐shelf materials, including balloons, sleeves, rubber bands, end caps, and zip ties. The pump and valve, which control the actuation of the McKibben actuator, can be precisely controlled by an embedded device such as an Arduino. These properties make McKibben actuators suitable as the actuation mechanism for PASTA.

**Figure 3:**
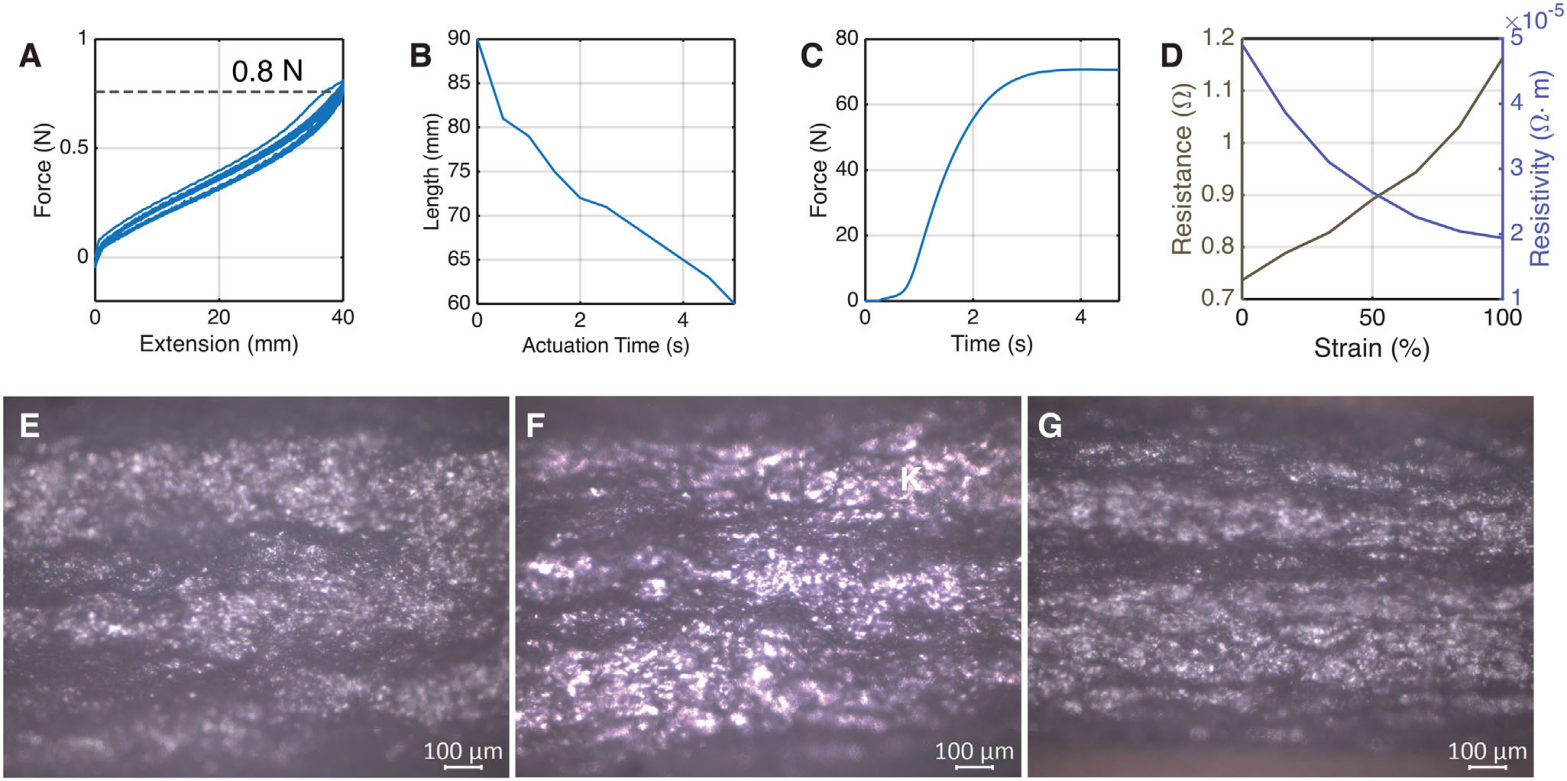
Material Characterization. A) The antenna branch shows a 0.8 N maximum force output for one antenna branch at 40 mm for 16 cycles. B) Actuation speed of the McKibben actuator at zero load. C) Force output of the McKibben actuator at zero strain. D) Resistance and calculated resistivity of the Ag‐EGaIn‐SIS trace of length 30 mm, thickness 0.08 mm, and width 0.5 mm. E) Confocal microscopy image of the embedded Ag‐EGaIn‐SIS trace inside silicone rubber, when no strain is applied. F) Confocal microscopy image of the embedded Ag‐EGaIn‐SIS trace inside silicone rubber, when 50% strain is applied. G) Confocal microscopy image of the embedded Ag‐EGaIn‐SIS trace inside silicone rubber, when 100% strain is applied.

PASTA's antenna branches and the McKibben actuators work collaboratively to achieve efficient actuation. Therefore, the mechanical performance of the two parts needs to correspond. **Figure** **3**A shows when the antenna branch is tested for 16 cycles with the extension from 0 to 40 mm, the tension increases nearly linearly from 0 N to 0.8 N. Figure 3B shows a McKibben actuator that is originally 90 mm long. After actuation, the length of the actuator decreases to 60 mm. Figure 3C shows the actuation speed of the actuator for the length to decrease from 90 to 60 mm is at a maximum of 5 seconds. The force output of the McKibben actuator is around 70 N, which is far stronger than the tension produced by the antenna. Therefore, a single actuator can quickly actuate the antenna to a contracted length of 30 mm.

To enable a high antenna efficiency during frequency reconfiguration over significant shape deformation, we select a conductive trace from a mixture of Silver powder (Ag SF91), eutectic Gallium–Indium alloy (EGaIn), and copolymer polystyrene‐block‐polyisoprene‐block‐polystyrene (SIS), shortened as Ag‐EGaIn‐SIS trace.^[^ ^68^ ^]^ We adapt the Ag‐EGaIn‐SIS trace instead of bulk EGaIn for three reasons. First, although EGaIn has a high fluidity, it has a high surface tension that can resist printing and wetting, making it difficult to infiltrate, bind tightly, or flow freely on a silicone rubber surface. At high strain, the EGaIn trace on a surface can easily get disconnected into islands where EGaIn droplets form. In contrast, the Ag‐EGaIn‐SIS trace is reported to remain robustly connected during as high strain as 500%, which meets our need in antenna reconfiguration.^[^ ^68^ ^]^ Second, Ag‐EGaIn‐SIS trace is a solid trace, which exhibits higher mechanical robustness compared to liquid EGaIn. Ag‐EGaIn‐SIS is reported to have the capability to self‐heal after experiencing high strain.^[^ ^69^ ^]^ In comparison, the flowing nature of EGaIn does not guarantee adequate mechanical robustness at high strain and has the risk of leaking and contamination once the seal experiences material defects or damages. Third, Ag‐EGaIn‐SIS traces are easier to fabricate using 3D printing, while EGaIn traces need to be constructed through micro‐tubing inside silicone rubber substrates. The ease of fabrication gives the antenna produced with Ag‐EGaIn‐SIS traces the potential to scale in production.

We then characterize the resistance and resistivity change of the Ag‐EGaIn‐SIS trace we use on the antenna. Figure 3D shows the change of resistance and resistivity when a strain is applied from 0% to 100%. The resistance increases from 0.74 Ω to 1.16 Ω when the trace length increases from 30 mm to 60 mm, with the calculated resistivity decreasing from 5× 10^−5^ Ω ·m to 2× 10^−5^ Ω ·m. We investigate the decrease in resistivity in microscopy images shown in Figures 3E‐G, where the figures show a microscopy image of the conductive trace at 0%, 50%, and 100% strain, respectively. It can be seen in Figure 3E that small silver flakes are visible at 0% strain while in Figure 3G, the silver flakes are shown to be fully infiltrated and wrapped by the EGaIn. Better infiltration can cause a decrease in the resistance between silver and EGaIn interfaces, which results in a decrease of resistivity globally. The overall decrease in resistivity also aligns with previous reports.^[^ ^68^ ^]^ The small change of resistance enables a smaller dissipation loss of the antenna when configured at different frequencies.

### 2.3 Frequency Reconfiguration

We evaluate the frequency response of PASTA when different branches are stretched. To compare the performance of PASTA, we take a baseline of a flexible patch antenna made according to the same technique in.^[^ ^51^ ^]^ We first characterize the return loss of the antenna, which is an efficiency indicator of a single antenna. We further compare the efficiency of the antenna when operating as a part of radio‐frequency (RF) chain by comparing the S21 and received signal strength (RSS) when the antenna is configured to different frequencies.

**Figure 4:**
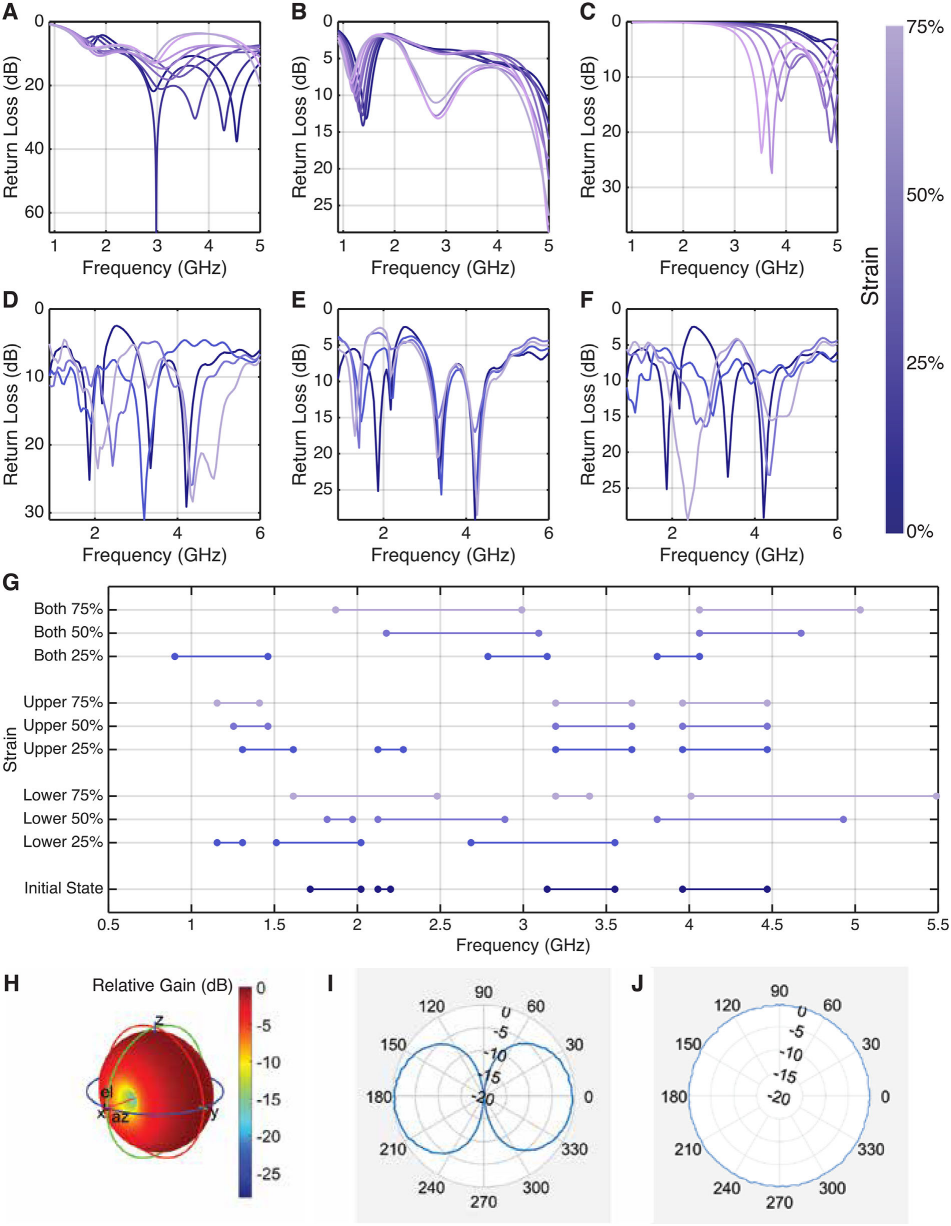
Antenna characterization. A) Simulated frequency when stretching the lower branch. B) Simulated frequency when stretching the upper branch. C) Simulated frequency when stretching the patch antenna. D) Measured frequency when stretching the lower branch, E) Measured frequency when stretching the upper branch. F) Measured frequency when stretching both branches. G) Effective operating frequency (with return loss < 10 dB) at each actuation state. H) Simulated 3D beam pattern, where each branch essentially acts as a dipole antenna and has a similar radiation pattern to dipole antennas. I) Measured radiation pattern on the *xOz* plane. J) Measured radiation pattern on the *yOz* plane.

We first characterize the frequency response of the antenna, showing that by shape deformation, the antenna can be reconfigured to efficiently operate at a wide range of different frequencies, spanning from 1 to 5 GHz. Return loss characterizes how efficiently an antenna transmits the signal. The larger the return loss is, the greater the percentage of input power is transmitted out by an antenna at a specific frequency. **Figure** **4**A,B shows the simulated return loss of the antenna when the upper and lower branch is stretched from 0% strain to 75% strain. As designed, during reconfiguration, the lower branch is designed to tune the operating frequency between 1.75 GHz to 5 GHz, and the upper branch is designed to tune the operating frequency between 1 and 1.75 GHz. Simulation results show that the antenna efficiency remains high, where the return loss is larger than 10 dB at the operating frequency during the reconfiguration process. We compare the simulation result with that of a flexible patch antenna shown in Figure 4C. When the patch antenna is stretched, the operating frequency only ranges between 4.5 and 5 GHz and 3 and 4 GHz, which only provides a 1.5 GHz operating frequency range during the reconfiguration process. It can therefore be inferred that PASTA provides 2.5 GHz more frequency coverage than a patch antenna.

Figure 4D–F shows the measured return loss of the lower branch, upper branch, and both branches of the antenna are actuated. Figure 4G shows the measured operating frequency range of the antenna at different actuation states. The operating frequency range is determined by the frequency at which the return loss is larger than 10 dB. To better depict the change in operating frequency, we name the three bands of our antenna cover simultaneously at the initial state (0% strain for both branches) as band A (1.75–2.25 GHz), band B (3.1–3.6 GHz), and band C (3.9–4.5 GHz), respectively.

When stretching the lower branch from 0% strain to 75% strain, band A first expands to 1.15–2 GHz, and then diminishes, while band B decreases and expands from 3.1–3.6 GHz to 1.6–2.5 GHz, and band C increases and expands from 3.9–4.5 GHz to 4–5.5 GHz. The observation also fits the trend we observe in the simulation shown in Figure 4A, where the band B around 3 GHz decreases and vanishes, and band C around 4.1 GHz increases to beyond 5 GHz.

When stretching the upper branch of the antenna from 0% strain to 75% strain, band B and band C remain almost the same, while band A slowly decreases from 1.75–2.25 GHz to 1.25–1.8 GHz. The decreasing trend also fits our observation from the simulation result in Figure 4B.

Finally, we characterize the change in operating frequency of the antenna when both branches are stretched from 0% strain to 75% strain. It can be seen that, similar to the phenomenon we observed for the lower branch, band A first expands to 0.8–1.48 GHz and then vanishes, while band B expands and decreases to 1.8–2.5 GHz, and band C expands and increases to 4.05–5.1 GHz. By combining the operating frequency we observed in Figure 4G, we reach a conclusion that our proposed antenna prototype covers the operating frequency from 900 MHz to 5 GHz as desired, which meets the operating frequency of radio frequency bands including cellular, WiFi, and Bluetooth.

One may note that although the overall trend of operating frequency change of the antenna fits between simulation and measurement, there are differences in the exact resonant frequency (where the return loss reaches a local maximum) and the return loss values. Multiple factors explain the difference. First, to measure the antenna return loss, we use the pneumatic actuator to reconfigure the antenna and measure the return loss when the pneumatic actuator is actuated at different states. However, it is worth noting that although the actuators are made of electrically insulating material, they still affect the S11 response as they can be viewed as a composite of the antenna substrate. Second, when the liquid metal‐based traces deform, their width, thickness, and resistivity change, which causes an impedance change in the traces. The change is difficult to capture exactly through theoretical computations and in simulation, and thus causes an offset between simulation and measurement. We hope that future research on the properties of liquid metal and liquid‐metal‐based conductive materials will improve our understanding of such materials and improve the accuracy of simulations. Third, 3D printing does not create an ideal trace that is homogeneous, flat, and smooth, which is modeled in the simulation software. Therefore, the microstructure of the traces that make up the antenna effects the S11 response, either changing the operating frequency due to a change in effective electrical length or changing the S11 values due to the difference in electrical impedance. Fourth, the antenna‐to‐SMA interface is an interface that connects flexible traces and rigid components. We use thin copper wires as the interface, with one side wrapped inside the flexible traces and strengthened via Silver‐epoxy, and the other side soldered on the SMA connector. While the interface is stable and well‐connected when the antenna is fabricated, when the shape of the antenna deforms, the interface between the flexible traces and the SMA connector changes, changing its reflection coefficient, which further affects the S11 readings.

We then show the radiation pattern of PASTA's antenna. Figure 4H shows the simulated directivity of the PASTA antenna, where it shows a radiation pattern similar to that of a monopole antenna. Further, we measure the antenna gain in the θ and ϕ direction in an anechoic chamber. Figure 4I shows the radiation pattern on the *xOy* plane, and Figure 4J shows the radiation pattern on the *xOz* plane. The measured radiation patterns also show that PASTA's antenna is an omni‐directional antenna similar to dipole and whip antennas.

**Figure 5:**
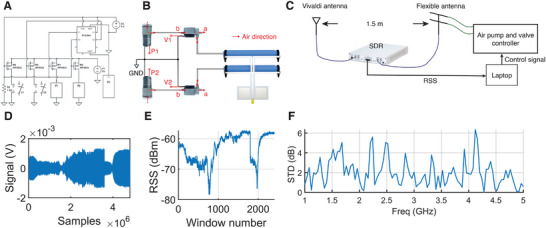
Self configuration of PASTA. A) The pump and valve are controlled through P‐MOS connected to an Arduino controller. A laptop sends control signals to control the on‐off of the valve and pump by the current received signal strength (RSS) values. B) Air flow diagram shows that the pump controls if air intake is enabled for the system, and valve controls whether the actuator is connected to the outside or the pump. Therefore, three modes are enabled. Actuation mode: actuator is connected to the pump and pump is on. Hold mode: actuator is connected to the pump and pump is off. Release mode: actuator is connected to the outside. C) Experiment setup where the transmitter and receiver are 1.5 m apart. D) Received signal with regard to time during the auto‐configuration process of PASTA. E) Received signal strength with regard to time during the auto‐configuration. F) Standard deviation (STD) of received signal strength in dB collected across three different samples.

To quickly adapt to different configurations when the operating frequency changes, PASTA achieves self‐configuration through a two‐staged algorithm. The first stage can be achieved offline, where a table is established to measure the antenna's performance across all frequencies at different actuation stages (time of actuation). When the antenna is configured to the frequency, it first configures to the actuation stage. The antenna further uses a second stage PID algorithm to fine‐tune and maintain its actuation stage to achieve higher RSS. This is achieved by using a PID algorithm and target at higher RSS than the current measurement, while controlling the open and close of the valve and air pump. **Figure** **5**A shows the control circuit diagram, and Figure 5B shows a diagram for the air flow's direction.

To study its self‐configuration capabilities, PASTA is tested as a receiver, and we evaluate the performance of PASTA with a rigid Vivaldi antenna and a flexible patch antenna. Figure 5C shows our testing setup. We use three samples for PASTA to average the result. The Tx and Rx antennas are placed 1.5 m apart in an office environment with a rich multipath and a relatively open lab environment to mimic an actual use case. Since we use a directional antenna as Tx, the multipath poses a similar effect on all receivers.

Figure 5D,E shows the received modulated signal and the corresponding received signal strength with regard to the time when the antenna experiences the two stages of the antenna configuration at 3 GHz. In the first step, the antenna goes through the configuration initialization step, where the first two inflation steps take 5 seconds each and the two deflation steps take 2 seconds each. Then the antenna maintains a high RSS by the second step, where RSS first quickly rises to a high value, then slowly rises in the refinement step and achieves maximum.

The repeatability of PASTA is shown by demonstrating the variance of RSS between the three antenna samples being tested. Figure 5G shows the variance of RSS across different frequencies for the three antenna samples. It can be seen that with our 3D‐printing‐based fabrication method, the quality and functionality of the soft antenna stay stable across multiple samples, with the average variance staying below 3 dB and the maximum variance staying below 6 dB.

**Figure 6:**
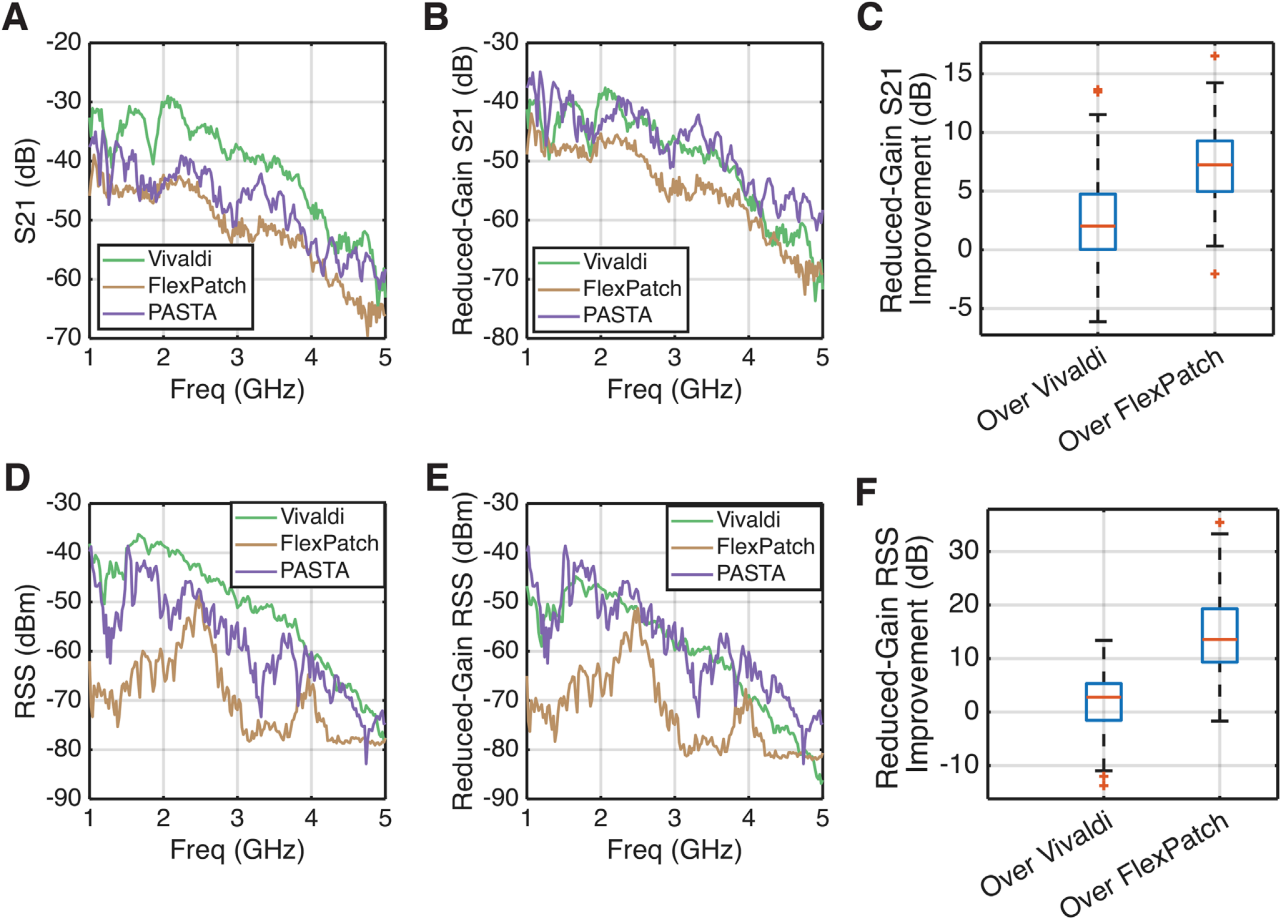
Performance of PASTA when working as a radio receiver. A) S21 Measurements with a Vivaldi antenna as the transmitter, and other antennas as receivers. B) By subtracting the S21 values from the directional gain, we obtain reduced‐gain S21 values which can be used to compare the radiation efficiency between three different receiver antennas. C) The improvement of PASTA over two baseline antennas in reduced‐gain S21. D) When using the transceiver chain inside a multipath‐rich indoor environment, we can mimic the received signal strength (RSS) by different antennas at different frequencies. E) The RSS is subtracted by the directional gain to show the overall antenna efficiency when applying to a practical‐mimicing environment. F) The RSS improvement of PASTA over two baseline antennas is approximately double the value of S21 improvement, since RSS is a power measure and S21 is a voltage measure.

We characterize the efficiency of PASTA over two baseline antennas: the Vivaldi antenna^[^ ^52^ ^]^ and a flexible patch antenna. In the test, we use a Vivaldi antenna as a transmitter, and compare the metrics of the radio‐frequency chain when three antennas are used as the receiver respectively. First, we take the S21 measurement inside an anechoic chamber, with the transmitter and receiver are placed 1.5 m apart, and the receiver and transmitter are aligned at the direction where directional gain is maximized. **Figure** **6**A shows the raw S21 measurement obtained when comparing the three antennas. It shows a reduction of S21 from lower frequencies and higher frequencies, where the bumps show the a frequency‐dependent performance change. The overall decreasing trend is likely due to the performance loss in both the Vivaldi antenna when moving from lower frequencies to higher frequencies, and the loss of the measurement hardware, such as the loss in the transmission lines and digital signal processing pipeline. To compare the radiation efficiency, we further subtract the directional gain of the antennas from the S21 values, and produce Figure 6B. It can be seen that PASTA provides a comparable S21 output with the Vivaldi antennas within the 1–4 GHz range and has a better performance in the 4–5 GHz range. PASTA surpasses the flexible patch antenna, which is designed at 3–5 GHz. Figure 6C shows the improvement of S21 of PASTA over Vivaldi and flexible patch antenna when the directional gain is reduced. It can be seen that in the 1–5 GHz designed operating range of PASTA, it has a 2 dB improvement in average reduced‐directivity S21 readings over Vivaldi antennas, and a 7.5 dB average improvement when compared to a flexible patch. This is because the flexible patch antenna is not designed to work in the 1‐4 GHz range.

In the second test, we record the received signal strength (RSS) of the transceiver pair, when the transceiver is placed inside a complex indoor environment with line‐of‐sight path and non‐line‐of‐sight paths. A number of non‐line‐of‐sight paths may affect the RSS readings at different frequencies due to fading effects. It is worth noting that in different environments, the exact readings at different frequencies can be different, while the overall trend should remain similar. Figure 6D shows the RSS reading when the three antennas are used as the receiver separately. The RSS reading from Vivaldi antenna surpasses the other two due to a higher directional gain. After subtracting the directional gain from the RSS readings, we obtain Figure 6E. Similar to the S21 result, the reduced‐gain RSS shows that PASTA is comparable to Vivaldi antennas in the 1–4 GHz range and surpasses Vivaldi antennas in the 4–5 GHz range. We observe an improvement in the reduced‐gain RSS of PASTA over Vivaldi to be 2.5 dB and over flexible patch antenna to be 13 dB, which is approximately double the value of the improvement of reduced‐gain S21.

Since the radiation efficiency is hard to measure directly, especially when a flexible antenna experiences multiple stages of reconfiguration, the metrics we use can be a reference for the antenna's radiation efficiency. These tests are intended to show that PASTA's efficiency toward practical use is comparable to state‐of‐the‐art rigid wideband antennas. However, several limitations still exist in our metric. First, return loss does not link directly with the radiation efficiency, as other loss such as dielectric loss and surface‐wave loss exists, especially for rigid wideband antennas. Second, the antenna's directional gain changes with frequencies. It can only be approximated, but can not be measured exactly at each frequency. Therefore, subtracting the S21 and RSS values by the directional gain may not provide exact radiation efficiency comparisons. Finally, the mismatch between the polarization direction of multiple antennas can affect the radiation performance between a radio‐frequency link, which further affects the S21 and RSS values.

PASTA is designed to function under long‐term usage. We perform cycle tests on 5 antenna samples. Each sample experiences 400 configuration cycles, where the antenna starts from configuration initialization, including two fully actuation and two fully de‐actuation steps, and then experiences a refinement procedure and a 10‐second position hold (Algorithm 2). The cycle test is done in an indoor environment with 40% humidity and 23°C room temperature. Among the 10 branches in the 5 samples, one balloon of two of the samples is damaged and causes leakage after 300 configuration cycles. The other 8 balloons remain working after the 400 cycles. Therefore, the mean time to failure (MTTF) is at least 380 cycles for a single actuator‐branch pair, and 360 cycles for a single antenna. Meanwhile, it is at low overhead to repair the damaged actuator. The damaged actuator can be removed and a new actuator can be attached to the branches using Silpoxy. Since the McKibben actuator has a simple construction, the mean time to repair (MTTR) of PASTA is low. Therefore, PASTA is robust to daily usage much like garments or other soft goods.

## 3 Discussion and Conclusion

This paper introduces PASTA, a pneumatically‐actuated, frequency‐tunable, flexible antenna that achieves 1–5 GHz operating frequency range through a multi‐branch antenna design. PASTA proposes impedance matching optimization to maximize overall antenna efficiency across a wide range of frequencies. A 3D‐printing‐based fabrication process is proposed for PASTA. Moreover, it utilizes a two‐stage configuration and control algorithm to maximize the antenna performance. Results show a 10 dB increment in average efficiency compared to a wideband antenna and a flexible patch antenna in the 1–5 GHz operating range, which corresponds to a 10 dB improvement in RSS and a 16 dB improvement when beamforming is applied.

It should be noted that PASTA is not aimed at substituting rigid antennas as many applications are well suited for this kind of traditional design. For instance, UWB antennas operate at a large range. Although they are not as efficient as PASTA, UWB usually sends a pulse where the antenna needs to work at a large bandwidth. In contrast, PASTA is a bandwidth‐limited antenna that is frequency tunable. Separately, PASTA is suitable for SDRs and wireless base stations where there is a stable power source and enough space for the antenna to be actuated. But for instances in which space is confined, e.g., a cellphone, since the working frequency bands are known, we can design a rigid antenna that matches well in these bands.

What this work unlocks is antenna designs that are lightweight, soft, and can operate over large frequencies which, in the future can be integrated well with soft robotic and wearable systems. More generally, the framework introduced here is generalizable for a whole host of other antenna designs and applications. In particular, this paper introduces stretchable two‐branch monopole antennas. There are other forms of antennas that act differently from microstrip antennas, e.g., waveguide antennas, coil antennas, etc. Future work can focus on generalizing this approach with different optimization methods to optimize frequency‐reconfigurable antennas of these different types. The concept of pneumatic actuation is also generalizable to other forms of flexible antennas. In this paper, as the baseline, we have explored combining pneumatic McKibben actuators with flexible patch antennas and measuring their performance. A similar process can be carried out for antennas that support frequency reconfiguration through linear shape deformation, including dipole and monopole antennas, Yagi–Uda antennas, and Helical antennas. The antennas can be reconfigurable using flexible circuits and substrates and attaching a pneumatic actuator along the direction where the strain occurs. In parallel, different actuation methods can be introduced to fit different antenna types. For example, aside from linear actuation, bending can also affect PASTA's frequency response and directivity. By combining linear actuation and bending, more complex antenna configurations are possible.

## 4 Experimental Section

### Antenna Optimization Implementation

Optimization using Eq. (3) is computationally inexpensive. We solve the problem by setting a large set of initial points. Our fabrication capability constrains the search space, e.g., the spacing and line width. We first use a non‐linear optimization heuristic (fmincon from Matlab) to find a parameter set. We then use the integrated gradient descent function in Ansys HFSS R2023 simulation software to find the refined result using Eq. (5).

### Antenna Substrate and Conductive Traces Fabrication

PASTA's antenna substrate is fabricated using Smooth‐On EcoFlex 00‐10 Silicone rubber due to its small Young's modules and shape‐deformable feature. This allows rapid actuation of the antenna with a small amount of force. The substrate is fabricated into 100 mm × 100 mm × 0.5 mm thin films using molding. The mold is fabricated using laser‐cut Acrylic sheets of thickness 0.5 mm. EcoFlex 00‐10 Part A and Part B are mixed in a 1:1 ratio and stirred thoroughly. It is then poured into the mold and degassed for 3 minutes. The silicone rubber cures at room temperature for 12 hours.

PASTA adapts a conductive elastic trace^[^ ^38^, ^63^, ^70^ ^]^ that serves as the stretchable antenna. The conductive trace remains conductive at a maximum stretch of 1000%, which corresponds to the breaking point of the silicone rubber, making our antenna extendable to a maximum of 10× frequency change for a single branch. The conductive elastic trace is fabricated by mixing 1.24 grams of SF91 Silver Flake (Ames–Goldsmith), 3 grams of EGaIn^[^ ^71^, ^72^ ^]^ (fabricated by heating Gallium 75%:25% Indium wt. at 200 degrees for 12 hours), and 1 gram of SIS (Copolymer polystyrene‐block‐polyisoprene‐block‐polystyrene.)‐Toluene solution (SIS 15%:85% Toluene wt., Sigma‐Aldrich). The solution is then mixed by a planetary centrifugal mixer (AR‐100; Thinky Corporation) at 3000 rpm for 3 minutes. It is then 3D printed on the silicone rubber substrate using a DIW 3D printer (System 30M Hyrel 3D) with a 25 Gauge Luer tip on a 5 mL syringe.

The conductive ink does not naturally bond with the silicone rubber substrate. To improve bonding, we select SF91 as the size of the Silver Flakes mixed inside the ink, as it may correspond to the surface roughness of the silicone rubber, and thus improve the bonding behavior.^[^ ^73^ ^]^ However, the conductive trace is still easy to peel off the silicone rubber surface. Therefore, we plasma treat the silicone rubber substrate at 1 atm Oxygen for 60 seconds (SPI Plasma Prep III). This allows negative ions to form on the surface of the silicone rubber substrate. When the ink is printed onto the surface while the ions are still present, the SIS is bonded to the surface by electrostatic force.^[^ ^74^, ^75^ ^]^ With such a treatment, the conductive traces adhere to the surface without being detached when the antenna is stretched.

We then add connectors to the antenna to enable a universal SMA connection. The insulation layers of 20 mm 30 Gauage copper wires are removed by scratching. They are then inserted into the designed ports on the printed ink pattern with the other ends staying outside. When the antenna is stretched, the copper forms an alloy with silver and EGaIn inside the conductive ink, which provides a low contact resistance.^[^ ^76^ ^]^ The antenna is put inside a fume hood at room temperature for 24 hours for the Toluene inside the conductive ink to evaporate. Finally, a 5 mm layer EcoFlex 00‐10 layer is deposited on top of the antenna to seal it. A small amount of Smooth‐On Silpoxy (a type of silicone rubber glue) is added to the contact points of the copper wires to strengthen them. The system is then set at room temperature for another 12 hours for the silicone rubbers to cure. The SMA connectors are soldered onto the copper wires outside to enable connection. We also use a small amount of Silpoxy to fix the SMA connectors on the antenna surface to prevent it from pulling the copper wires from the conductive traces.

### Pneumatic Actuators

The Mckibben actuators are fabricated using balloons and plastic sleeves. They are constructed by cutting a piece of nylon mesh (Flexo Fluorescent 3/8″) and a party balloon (Kuulbela), each between 10 and 13 cm in length. The balloon is carefully inserted into the nylon mesh. One end of this assembly is secured to a barbed end cap using a rubber band and a zip tie to ensure a tight seal. The opposite end is similarly attached to a barbed connector, again using a rubber band and a zip tie, to facilitate the passage of air.

### System Packaging

In order to make a minimized antenna, instead of using actuators on the side pulling the antenna branches, we pre‐stretch the antenna branches and attach them to the actuators. For attachment, the excess substrate is cut out, leaving only thin branches. This can reduce the tension force produced by the stretched branches so that they do not bend the McKibben actuators. The branches are then stretched to 175%, which corresponds to the contraction ratio of the actuators. Next, the edges of the branches are fixed to the edges of the actuators by Silpoxy. During the curing process of Silpoxy, a rubber band is used to fix the stretching state of the branches. The Silpoxy is cured for 12 hours and the rubber bands are cut off before the antenna is ready to use.

### Frequency Measurements

Simulated results are acquired using Ansys HFSS 2022 simulation. Measured results are acquired by actuating the actuator to different states and return loss values are measured by a VNA (Keysight N9916A Microwave Analyzer). The experiment is done inside an anechoic chamber (Sub 6 GHz).

### Directivity Measurements

Directivity is measured by an antenna measurement system (DAMS X100 Antenna Measurement System, Anritsu MS2028C Vector Network Analyzer) inside an anechoic chamber (Sub 6 GHz).

### Form Factor Measurements

Form factor is measured by a caliper. Other form factors are acquired through data sheets and paper descriptions.^[^ ^28^, ^51^, ^52^, ^53^ ^]^ PASTA's form factor is similar to a flexible dipole antenna or patch antenna, and much smaller than common wideband antennas.

### Mechanical Evaluation

Two sides of the antenna branch or the actuator are clamped on two ends of a material testing machine (Instron Model 5969). For a stretch test, the antenna branch is stretched at rate 0.5 mm s^−1^ until a total extension of 40 mm. For a iso‐strain test, the actuator is fixed at position and pumped with a ZR370‐02PM air pump with 5 V input voltage.

### Resistance and Resistivity Measurements

Resistance and resistivity of a conductive trace of initially 30 mm length, 0.4 mm thickness, and 5 mm width. The resistance is measured through a DC power supply (Rigol DP832A) with integrated high‐precision voltage and current meters. The voltage is set to 0.5 V and current is recorded for different strain. Resistance is calculated through dividing the voltage by the current. Resistivity is calculated by assuming the volume does not change for the conductive trace, where for each strain, cross‐sectional area is calculated by dividing the volume by the length. Resistivity is calculated by dividing the resistance by the length times the cross‐sectional area.

### Confocal Microscope Imaging

Optical images were acquired using a confocal microscope (Zeiss LSM 900) equipped with a 10X objective.

### Self‐Configuration Test

To study its self‐configuration capabilities, PASTA is tested as a receiver. The transmitter is a Vivaldi wideband antenna.^[^ ^52^ ^]^ All antennas tested are aligned with the maximum directional gain. We use a USRP X310 SDR to generate single‐tone waves of <100 kHz bandwidth at uniformly sampled 401 frequency points from 1 GHz to 5 GHz.

### S21 Measurements

S21 are measured inside anechoic chamber with a LibreVNA connected to a laptop with automated recording scripts. The transceiver pair is placed 1.5 meters apart, line‐of‐sight, adjusted to the maximum directional gain.

### RSS Measurements

Received signal strength (RSS) is calculated by taking the raw signal in a time window, take the average (denoted by $\bar{V}$, and calculate by RSS = $20\log_{10}\left(\bar{V}\right)$.

### Beamforming

To enable beamforming, we use two identical PASTA samples that are separated apart by 15 cm, which is half of the wavelength of the 1 GHz wave. Then then use the same configuration as the self‐configuration test to acquire the signals and calculate RSS.

### Control Circuit

PASTA is able to automatically adjust its configuration to the operating frequency. The signal quality is evaluated by the received signal strength (RSS), which can be continuously monitored by the receiver. To quickly achieve the desired actuation state, we develop a two‐stage optimization process. The first stage is a pre‐configuration step done offline. In the first stage, a sub‐optimal actuation state for each frequency is explored. The result is universal for all the antennas and can be done offline. The small individual differences of each antenna in fabrication and the environment such as air pressure and temperature can cause a deviation from the optimal configuration. Therefore, in the second stage, an online refinement algorithm is proposed to tune the antenna performance through the RSS feedback.

The two branches of the antenna are designed to be responsible for different frequency ranges. Meanwhile, the two branches also couple with each other which affects the overall input impedance of the antenna. Therefore, to achieve optimal efficiency at each operating frequency, we use two separate actuation chains for both actuators. The control circuit (Figure 5A) is composed of four P‐MOSFETs (IRF9530) control pumps (ZR370‐02PM) and valves (FA0520E). The ON/OFF status of the pumps and the OPEN/CLOSE status of the valves are determined by their input voltage. The airflow diagram (Figure 5B) shows that when the voltage supply for the valve is high, airflow a is enabled and airflow b is disabled, causing the air to flow out of the actuator. We mark it as OPEN. On the other hand, when the voltage supply is low, airflow a is disabled, and airflow b is enabled, causing the air to flow inside the actuator from the pump. We mark it as CLOSED. Note that when the valve is CLOSED, *air will not flow out of the actuator* even when the pump is OFF, because the pump only enables single‐direction airflow. This enables our system to stay at a specific actuation status without a consistent power supply.

### Offline Configuration Initialization

When the antenna system starts for the first time, the system leverages a one‐time configuration that uses an actuation state sweep to find an optimal actuation range. The result of the one‐time configuration for each frequency point can be saved for future use. We uniformly sample 401 frequency points from 1 to 5 GHz. Since there are two actuators, the search space for the possible actuation combinations is too large, where we need to maintain one actuator at each actuation state and fully actuate the other actuator, which can take tens of rounds for each possible frequency. Therefore, it is impossible to attempt all actuation configurations. Moreover, even if we iterate over all actuation configurations offline, the offset in the antennas and the operation environment would require further online refinement as well. Instead, we leverage an observation that separate branches dominate the response at different frequency ranges. In such a process, for each frequency, the optimization can be simplified to finding the configuration of the branch that mainly decides the frequency response. Meanwhile, the other branch which only affects the impedance, will only be tuned in the online refinement step. Therefore, we separately actuate two branches to find the optimal actuation configuration. The process includes four stages described in Algorithm 1. The flow of the four stages covers the situations when one branch actuates from zero to maximum, while the other branch stays at zero or maximum actuation.

At the beginning of initialization, we turn all valves open and all pumps off to release the air inside the system (Lines 1). In each actuation stage, we record the RSS collected throughout different actuation states, aggregate the results, and aim to find the best actuation state among the states. In the first stage, antenna 1 is actuated to the maximum in 6 seconds, while antenna 2 remains unactuated (Lines 5). In the second stage, antenna 2 is actuated to the maximum in 6 seconds, while antenna 1 remains fully actuated (Lines 7). At the end of the second stage, antennas 1 and 2 are both fully actuated. Antenna 1 is then deactuated in 2 seconds (Lines 9). Finally, in the fourth stage, antenna 2 is deactuated (Lines 11). A thread is opened to record the RSS during the process (Line 3). At the end of each stage, we take the average RSS of a 10 millisecond time window (Lines 14‐17). We then find the actuation point according to the maximum windowed RSS values (Line 18). The actuation state refers to the duration at which the pump needs to be kept on (Line 19). Finally, we compare the RSS from the four stages, and record the configuration that provides the best RSS values (Line 21). It provides an estimate for the optimal configuration, which we can then tune for the next refinement step.

Algorithm 1 **Configuration Initialization.**

**Table:** 

1:	Valve_1, 2_ ←OPEN, Pump_1, 2_ ←OFF
2:	**for** Stage = 1; Stage ⩽ 4; Stage++ **do**
3:	***Thread 1***: Record RSS of length *N* in vector R.
4:	**if** Stage == 1 **then**
5:	***Thread 2***: Valve_1_ ←CLOSED, Pump_1_ ←ON;
6:	**else if** Stage == 2 **then**
7:	***Thread 2***: Valve_2_ ←CLOSED, Pump_2_ ←ON;
8:	**else if** Stage == 3 **then**
9:	***Thread 2***: Valve_1_ ←OPEN, Pump_1_ ←OFF;
10:	**else if** Stage == 4 **then**
11:	***Thread 2***: Valve_2_ ←OPEN, Pump_2_ ←OFF;
12:	**end if**
13:	**join** *Thread 1, Thread 2*
14:	// average RSS in a time window
15:	**for** *w* = 1; *w* < *w*_max _; *w* + + **do**
16:	$\bar{R}\left[w\right]\leftarrow \mathrm{mean}\left\{R[\left(w-1\right)\frac{N}{w_{\max}}+1:w\frac{N}{w_{\max}}]\right\}$
17:	**end for**
18:	*w*_*o*_[Stage]←argmaxR
19:	Actuation[Stage]←Time according to *w*_*o*_.
20:	**end for**
21:	$S_{o}\leftarrow \arg\max w_{o}$
22:	**set** system to Actuation[*S*_*o*_]

### Refining Optimal Configuration

We configure the antenna to the optimal performance with two branches. one branch (denoted as the main branch) controls the resonate frequency, and the other branch (denoted as the side branch) affects the impedance, which also affects the antenna efficiency. In the initial configuration step, we have configured the main branch. In the refinement step, we tune the branches to improve impedance matching and compensate for antenna fabrication differences and environmental differences. Since the RSS values are always available during transmission, we use an online feedback control loop. The search space for the main branch is confined around the result provided by the initialization step.

We use a proportional‐integral‐derivative (PID) auto‐configuring algorithm that refines the configuration to a higher RSS, described in Algorithm 2. The PID‐based optimization is based on the observation that the response frequency and impedance change are linear to the actuation state of the branches. Therefore, to set an RSS value that is higher than the current RSS value, the PID controller automatically tunes the system to a better configuration so that the higher RSS value is achieved at the best attempt. The PID controller has three parameters, *K*_*P*_, *K*_*I*_, *K*_*D*_, corresponding to the coefficients of PID terms respectively. The PID controller can be formally expressed as follows:

(6) $$A\left(t\right)=K_{P}R_{e}\left(t\right)+K_{I}\int_{0}^{t}R_{e}\left(\tau \right)d\tau +K_{D}\frac{dR_{e}\left(t\right)}{dt}\;,$$

where A(t) is a 2D vector that corresponds to two actuators, and *R*_*e*_(*t*) is the difference between the current RSS and the target RSS per time frame. *A*(*t*) > *A*_0_ symbolizes actuation, and *A*(*t*) < −*A*_0_ symbolizes de‐actuation. The parameter *A*_0_ is a threshold that we set as no action to prevent overshoot.

We set two parameters, RSS per‐step improvement *p* and decaying rate ν (Line 1). A thread listens to the RSS during communication (Line 2). The PID controller optimization is done 10 recurrent times, each time the objective decays with a decaying rate ν. Here, we denote the optimal RSS we can get at step *i* with the value *w*_*i*_. Then in step *i* + 1, we aim at improving *w*_*i*_ to *w*_*i*_ × *p* × ν^*i*^ as the best effort (Line 5). We then use the PID controller to try to get an improved actuation result according to the new RSS target (Lines 6‐10). The PID controller may produce unusual actuation orders for unachievable. In such a case, we reduce the target RSS, represented by the decaying factor. The process is done in 5 iterations. A PID algorithm is then used to maintain the optimal RSS to avoid small air leakage from the system. To save power, the PID is tuned only occasionally, and during rest time, the valve remains closed.

### PID Parameter Tuning

Tuning the parameter for the PID controller is critical for its performance. A large *K* parameter set can cause overshoot which destroys the stability of control. If the *K* parameters are too small, the convergence can be too slow. Note that with different input power, actuators, and antenna lengths, the *K* parameters can be set differently. We adjust the parameters by hand according to the previous observations, setting a lower *K* parameter from the beginning, and slowly increasing *K* until we observe oscillation happens in the system. We then decrease *K* to which the oscillation is removed in the 1‐second time window.

Algorithm 2 **PID actuation fine tuning.**

**Table:** 

1:	*p* ← 5%, ν ← 0.5
2:	***Thread 1***: Listening RSS consistently.
3:	**for** (*i* = 0; *i* < 5; *i* + +) **do**
4:	*R*(*t*) ← RSS measurement
5:	*w* ← (1 + *p*ν^*i*^)*R*(*t*)
6:	**while** *t* < 1 second **do**
7:	*R*(*t*) ← RSS measurement
8:	A(t)←PID(R(t)−w)
9:	Actuate according to A(t).
10:	**end while**
11:	**end for**
12:	**while** *t* < 1 second **do**
13:	*R*(*t*) ← RSS measurement
14:	A(t)←PID(R(t)−w)
15:	Actuate according to A(t).
16:	**end while**

### Control Implementation

The control signal is provided by the PC to the Arduino, which further transmits high/low voltage to the PMOS which regulates the ON/OFF behavior of the pumps and valves. On the PC side, a main program talks to the Arduino program using serial UART. The main program is written in C++. RSS signal is acquired through a USRP SDR (X310) with two UBX daughterboards responsible for Tx and Rx respectively.

### Statistical Analysis

Simulation data are produced by one‐time run in the simulation software. Experiments, if not specified otherwise, are tested and averaged on three independent samples. The red line in box plot shows average value, blue box shows 25‐75% confidence interval, dashed line shows 5‐95% confidence interval, and red markers show outliers. No pre‐processing of the results are included.

## Conflict of Interest

The authors declare no conflict of interest.

## Supporting information

Supporting Information

## Data Availability

The data that support the findings of this study are openly available in Dropbox at https://www.dropbox.com/scl/fo/3bb1hkkoyizxkn0ko7hit/APvutysVKTZ_TwmYANflgR0?rlkey=c7zl4u632cytwzm1s9v2qoric&dl=0, reference number 0.

## References

[1] T. Ulversoy , IEEE Commun. Surv. Tutor. 2010, 12, 531.

[2] Y. Xu , R. K. Amineh , Z. Dong , F. Li , K. Kirton , M. Kohler , Sensors 2022, 22, 6455. 36080914 10.3390/s22176455 PMC9459827

[3] W. Jeong , J. Jung , Y. Wang , S. Wang , S. Yang , Q. Yan , Y. Yi , S. M. Kim , in MobiCom 2020, pp. 1–14.

[4] A. M. Wyglinski , D. P. Orofino , M. N. Ettus , T. W. Rondeau , IEEE Commun. Mag. 2016, 54, 68.

[5] M. A. Ramos , R. Camacho , P. A. Buitrago , R. D. Urda , J. P. Restrepo , in *Feduc*, Volume 8, Frontiers Media SA 2024, 1228610.

[6] S. O. Murphy , C. Sreenan , K. N. Brown , in VTC , IEEE 2019, pp. 1–6.

[7] P. I. Theoharis , R. Raad , F. Tubbal , M. U. A. Khan , S. Liu , J‐MASS 2020, 2, 10.

[8] J. Abraham , K. Venusamy , A. Judice , H. Shaik , K. Suriyan , IJRES 2023, 2089, 4864.

[9] R. D. Raut , K. D. Kulat , in ICMSAO , IEEE 2011, pp. 1–8.

[10] F. K. Jondral , IEEE Wirel. Commun. 2007, 14, 28.

[11] T. Kim , W. Lee , in INFOCOM , IEEE 2019, pp. 262–270.

[12] S. Li , H. Zheng , C. Zhang , Y. Song , S. Yang , M. Chen , L. Lu , M. Li , in NSDI 2022, pp. 913–928.

[13] Z. Yang , Q. Huang , Q. Zhang , in MobiCom 2017, pp. 356–367.

[14] I. K. Jain , R. Subbaraman , D. Bharadia , in MobiCom 2023, pp. 1–3.

[15] R. Zhao , T. Woodford , T. Wei , K. Qian , X. Zhang , in MobiCom 2020, pp. 1–14.

[16] D. Huang , R. Nandakumar , S. Gollakota , in SenSys 2014, pp. 266–279.

[17] J. Guan , A. Paidimarri , A. Valdes‐Garcia , B. Sadhu , in RFIC , IEEE 2020, pp. 147–150.

[18] S. Zheng , Z.‐Y. Zhang , X. Chen , A. A. Kishk , IEEE Trans. Antennas Propag. 2022, 70, 7118.

[19] G. A. Hofbauer , in IEEE MTT‐S Int. Microw. Symp. Dig , IEEE 2005, pp. 551–554.

[20] M. Mobrem , C. Spier , in AMS 2012, pp. 127–140.

[21] M. E. McEachen , in AIAA Spacecraft Structures Conference 2018, 1945.

[22] A. Mansour , A. F. Tayel , A. Khames , M. Azab , S. I. Rabia , N. Shehata , AEU ‐ Int. J. Electron. Commun. 2019, 102, 25.

[23] D. Corzo , G. Tostado‐Blázquez , D. Baran , Front. Electron. 2020, 1, 594003.

[24] P. Wang , M. Hu , H. Wang , Z. Chen , Y. Feng , J. Wang , W. Ling , Y. Huang , Adv. Sci. 2020, 7, 2001116. 10.1002/advs.202001116 PMC7578875 33101851

[25] N. Matsuhisa , X. Chen , Z. Bao , T. Someya , Chem. Soc. Rev. 2019, 48, 2946. 31073551 10.1039/c8cs00814k

[26] S. Yao , Y. Zhu , Adv. Mater. 2015, 27, 1480. 25619358 10.1002/adma.201404446

[27] Z. Xie , R. Avila , Y. Huang , J. A. Rogers , Adv. Mater. 2020, 32, 1902767. 10.1002/adma.201902767 31490582

[28] L. H. Blumenschein , L. T. Gan , J. A. Fan , A. M. Okamura , E. W. Hawkes , IEEE RA‐L 2018, 3, 949.

[29] L. Song , W. Gao , C. O. Chui , Y. Rahmat‐Samii , IEEE Trans. Antennas Propag. 2019, 67, 2886.

[30] M. Kelley , C. Koo , H. McQuilken , B. Lawrence , S. Li , A. Han , G. Huff , Electron. Lett. 2013, 49, 1370.

[31] L. Shao , X. Tang , Y. Yang , D. Wei , Y. Lin , G. He , D. Wei , J. Phys. D 2022, 55, 195301.

[32] Y. Liu , Q. Wang , Y. Jia , P. Zhu , IEEE Trans. Antennas Propag. 2020, 68, 7630.

[33] X. Chen , K. Li , IEEE Antennas Wirel. Propag. Lett. 2022, 22, 960.

[34] H. R. Khaleel , H. M. Al‐Rizzo , D. G. Rucker , S. Mohan , IEEE Antennas Wirel. Propag. Lett. 2012, 11, 564.

[35] H. Kanaya , S. Tsukamaoto , T. Hirabaru , D. Kanemoto , R. K. Pokharel , K. Yoshida , IEEE Microw. Wirel. Compon. Lett. 2013, 23, 164.

[36] B. Yao , X. Xu , Q. Zhang , H. Yu , H. Li , L. Ren , S. Perini , M. Lanagan , Q. Wang , H. Wang , Mater. Lett. 2020, 270, 127727.

[37] Y.‐W. Wu , S. Alkaraki , S.‐Y. Tang , Y. Wang , J. R. Kelly , Proc. IEEE 2023, 111, 955.

[38] M. R. Vinciguerra , D. K. Patel , W. Zu , M. Tavakoli , C. Majidi , L. Yao , ACS Appl. Mater. Interfaces 2023, 15, 24777. 37163362 10.1021/acsami.2c23028 PMC10214374

[39] C. Ohm , M. Brehmer , R. Zentel , in Liquid Crystal Elastomers: Materials and Applications 2012, pp. 49–93.

[40] R. S. Kularatne , H. Kim , J. M. Boothby , T. H. Ware , J. Polym. Sci. B 2017, 55, 395.

[41] H. Jiang , C. Li , X. Huang , Nanoscale 2013, 5, 5225. 23648966 10.1039/c3nr00037k PMC3697106

[42] H. Rodrigue , W. Wang , M.‐W. Han , T. J. Kim , S.‐H. Ahn , Soft Robotics 2017, 4, 3. 29182099 10.1089/soro.2016.0008

[43] J. Mohd Jani , M. Leary , A. Subic , J. Intell. Mater. Syst. Struct. 2017, 28, 1699.

[44] J.‐H. Youn , S. M. Jeong , G. Hwang , H. Kim , K. Hyeon , J. Park , K.‐U. Kyung , Appl. Sci. 2020, 10, 640.

[45] X. Cao , M. Zhang , Z. Zhang , Y. Xu , Y. Xiao , T. Li , Acta Mech. Solida Sin. 2019, 32, 566.

[46] A. O'Halloran , F. O'malley , P. McHugh , J. Appl. Phys. 2008, 104, 7.

[47] H. Chen , L.‐x. Cai , Acta Mater. 2016, 121, 181.

[48] G. Bradley , P. Chang , G. Mckenna , J. Appl. Polym. Sci. 2001, 81, 837.

[49] H. Kaupp , IEEE Trans. Elec. Comput. 1967, 185.

[50] Y. Chen , Y. Chi , J. Fan , C. Ma , Math. Program 2019, 176, 5. 33833473 10.1007/s10107-019-01363-6 PMC8025800

[51] M. R. Ramli , S. Ibrahim , Z. Ahmad , I. S. Z. Abidin , M. F. Ain , ACS Appl. Mater. Interfaces 2019, 11, 28033. 31314485 10.1021/acsami.9b07671

[52] RFSpace, Tsa 900 uwb antenna, http://rfspace.com/RFSPACE/Antennas_files/TSA900.pdf, 2024.

[53] RFElements, Starterhorn 30‐degree usma, https://rfelements.com/products/starterhorn/starterhorn‐30‐usma/technical‐data, 2024.

[54] C. Tawk , G. Alici , Adv. Intell. Syst. 2021, 3, 2000223.

[55] M. S. Xavier , C. D. Tawk , A. Zolfagharian , J. Pinskier , D. Howard , T. Young , J. Lai , S. M. Harrison , Y. K. Yong , M. Bodaghi , et al., IEEE Access 2022, 10, 59442.

[56] H. I. Ali , S. Noor , S. Bashi , M. H. Marhaban , AJBAS 2009, 3, 440.

[57] O. Kuksenok , P. Dayal , A. Bhattacharya , V. V. Yashin , D. Deb , I. C. Chen , K. J. Van Vliet , A. C. Balazs , Chem. Soc. Rev. 2013, 42, 7257. 23370524 10.1039/c3cs35497k

[58] H.‐J. Schneider , K. Kato , R. M. Strongin , Sensors 2007, 7, 1578. 19606275 10.3390/s7081578 PMC3814870

[59] X. Zhu , Y. Hu , G. Wu , W. Chen , N. Bao , ACS Nano 2021, 15, 9273. 34018737 10.1021/acsnano.1c02356

[60] D. K. Patel , X. Huang , Y. Luo , M. Mungekar , M. K. Jawed , L. Yao , C. Majidi , Adv. Mater. Technol. 2023, 8, 2201259.

[61] Z. J. Patterson , D. K. Patel , S. Bergbreiter , L. Yao , C. Majidi , Soft Robotics 2023, 10, 292. 35852561 10.1089/soro.2022.0003

[62] L. Plottel , R. Desatnik , D. K. Patel , P. LeDuc , C. Majidi , IEEE Robot. Automat. Lett. 2025, 10, 7579.

[63] Z. Li , G. Olson , D. K. Patel , L. Yao , C. Majidi , Adv. Intell. Syst. 2024, 6, 2200402.

[64] S. Xu , C. M. Nunez , M. Souri , R. J. Wood , Sci. Robot. 2023, 8, eadd4649. 37343077 10.1126/scirobotics.add4649

[65] M. Duduta , E. Hajiesmaili , H. Zhao , R. J. Wood , D. R. Clarke , PNAS 2019, 116, 2476. 30679271 10.1073/pnas.1815053116 PMC6377461

[66] A. Poulin , S. Rosset , H. R. Shea , Appl. Phys. Lett. 2015, 107, 24.

[67] C. A. Manion , D. K. Patel , M. Fuge , S. Bergbrieter , in International Conference on Intelligent Robots and Systems (IEEE/RSJ) , 2018.

[68] P. A. Lopes , D. F. Fernandes , A. F. Silva , D. G. Marques , A. T. de Almeida , C. Majidi , M. Tavakoli , ACS Appl. Mater. Interfac. 2021, 13, 14552. 10.1021/acsami.0c22206 33689286

[69] Y. Song , Z. Li , M. Zadan , J. Wang , S. Kumar , C. Majidi , Nat. Commun. 2025, 16, 7292. 40774948 10.1038/s41467-025-62313-9 PMC12332080

[70] M. Zadan , A. Wertz , D. Shah , D. K. Patel , W. Zu , Y. Han , J. Gelorme , H. J. Mea , L. Yao , M. H. Malakooti , S. H. Ko , N. Kazem , C. Majidi , Adv. Funct. Mater. 2024, 34, 2404861.

[71] M. Zadan , D. K. Patel , A. P. Sabelhaus , J. Liao , A. Wertz , L. Yao , C. Majidi , Adv. Mater. 2022, 34, 2200857. 10.1002/adma.202200857 35384096

[72] M. J. Ford , D. K. Patel , C. Pan , S. Bergbreiter , C. Majidi , Adv. Mater. 2020, 32, 2002929. 10.1002/adma.202002929 33043492

[73] W. Zu , Y. Ohm , M. R. Carneiro , M. Vinciguerra , M. Tavakoli , C. Majidi , Adv. Mater. Technol. 2022, 7, 2200534.

[74] T. Yamamoto , Surf. Interface Anal. 2013, 45, 817.

[75] J. Roth , V. Albrecht , M. Nitschke , C. Bellmann , F. Simon , S. Zschoche , S. Michel , C. Luhmann , K. Grundke , B. Voit , Langmuir 2008, 24, 12603. 18828614 10.1021/la801970s

[76] K. B. Ozutemiz , J. Wissman , O. B. Ozdoganlar , C. Majidi , Adv. Mater. Interfaces 2018, 5, 1701596.

