# Supplementary material for: Pneumatically‐Actuated Liquid Metal‐Based Frequency Reconfigurable Antenna
Source: Adv Sci (Weinh). 2025 Dec 12;13(5):e12996. doi: 10.1002/advs.202512996 (PMC12850249; doi:10.1002/advs.202512996)
Supplement: Supplementary file 1 — Supporting Information [file ADVS-13-e12996-s001.pdf]

Supplementary Materials

Actuatable Wide Bandwidth Liquid Metal Based Software Tunable Antennas

Yiwen Song, Aditya Bharambe, Dinesh K. Patel, Barbara Zhuo, Mason Zadan, Carmel Majidi*, Swarun Kumar**

Y. Song, B. Zhuo, Prof. C. Majidi, and Prof. S. Kumar
Department of Electrical and Computer Engineering, Carnegie Mellon University
5000 Forbes Avenue, Pittsburgh, PA, USA. 15213.
A. Bharambe, Dr. D. K. Patel, Dr. M. Zadan, and Prof. C. Majidi
Department of Mechanical Engineering, Carnegie Mellon University
5000 Forbes Avenue, Pittsburgh, PA, USA. 15213.
Dr. M. Zadan
Current Affiliation: Koch Institute for Integrative Cancer Research, Massachusetts Institute of Technology
77 Massachusetts Avenue, Cambridge, MA, USA. 02139.

Contents

1 Mechanical Testing **4**

2 Testing Setup **5**

List of Figures

S1 Mechanical testing of the antenna. (A) Testing setup of the antenna branch. (B) A cyclic extension test of the antenna branch. (C) Actuation of the McKibben actuator. 4
S2 Setup of the configuration experiment 5

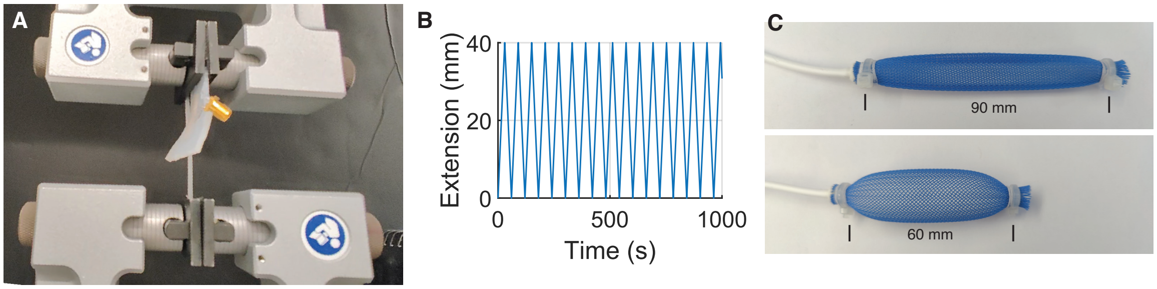

Figure S1: Mechanical testing of the antenna. (A) Testing setup of the antenna branch. (B) A cyclic extension test of the antenna branch. (C) Actuation of the McKibben actuator.

1 Mechanical Testing

Mechanical characterization of the antenna actuator and branch is conducted on a material testing machine (Instron; Model 5969). Figure S1A shows the testing setup of the mechanical test, where the branch of the antenna or the actuator are mounted on the grippers. Figure S1B shows the extension of the McKibben actuator during 10 actuation-deactuation cycles, where Figure S1C shows the testing setup of the cycle test of the pneumatic actuator. During the cycle test, one end of the McKibben actuator is mounted on a table with hot glue, where the other end is released free. We use video post processing to determine the length of the actuator during the actuation process and plot the extension-time plot.

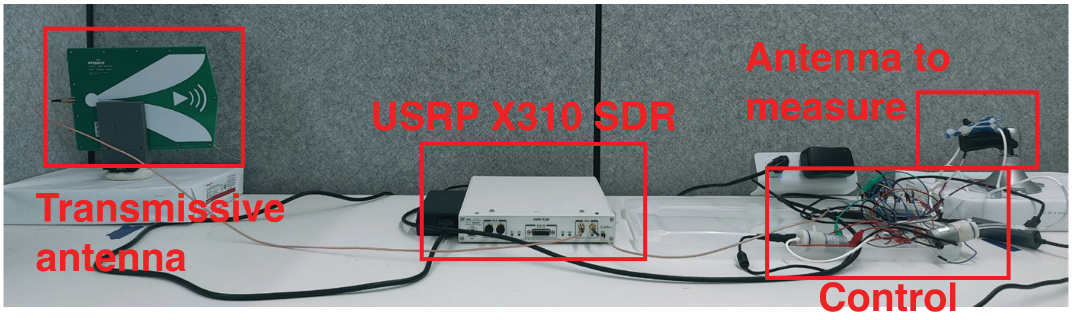

Figure S2: Setup of the configuration experiment

2 Testing Setup

Figure S2 shows the setup of our antenna self-configuration evaluation. The signal is generated by a USRP X310 software-defined radio (SDR), which is further controlled by a laptop. Two antennas are connected to the SDR. We use one UWB antenna as the transmitter antenna, and test the antenna's performance at its best direction.
